# Mental health literacy survey of non-mental health professionals in six general hospitals in Hunan Province of China

**DOI:** 10.1371/journal.pone.0180327

**Published:** 2017-07-05

**Authors:** Qiuxia Wu, Xiaoyang Luo, Shubao Chen, Chang Qi, Jiang Long, Yifan Xiong, Yanhui Liao, Tieqiao Liu

**Affiliations:** 1 Mental Health Institute of the Second Xiangya Hospital, Central South University, The China National Clinical Research Center for Mental Health Disorders, National Technology Institute of Psychiatry, Key Laboratory of Psychiatry and Mental Health of Hunan Province, Changsha, Hunan, People’s Republic of China; 2 Female Psychiatric Ward, the First Psychiatric Hospital of Hengyang City, Hengyang, Hunan, People’s Republic of China; 3 Laboratory for Experimental Psychopathology, Psychological Science Research Institute, Université Catholique de Louvain, Louvain-la-Neuve, Belgium; University of Texas Health Science Center at San Antonio Cancer Therapy and Research Center at Houston, UNITED STATES

## Abstract

**Background:**

Mental illness has brought great economic burden related to misdiagnosis by non-mental health professionals in general hospitals. The aim of this study was to explore non-mental health professionals’ conceptions related to the identification of mental illness and perceived treatments, first aid and prognosis.

**Methods:**

In 2014–2015, we presented 1123 non-mental health professionals from six general hospitals in Hunan Province with one of three vignettes describing a person with schizophrenia, depression, or generalized anxiety disorder. Identification rates, beliefs about various interventions, best methods, and the prognosis with or without treatment were measured.

**Results:**

Less than 60% of the non-mental health professionals could identify the mental disorders correctly. Psychiatrists and psychologists were considered to be the people who would be most helpful in all vignettes. Over 70% of participants identified the correct medication for each vignette. Participants gave higher ratings to lifestyle interventions than to psychological and medical interventions, especially in the depression and generalized anxiety disorder vignettes. For the question about how the person could best be helped, about half of the participants rated listening or talking with the person more highly than accompanying the person to professional help or encouraging the person to visit a psychiatrist or psychologist. Participants believed that, with professional help, the people in the vignettes would fully recover but that problems would probably reoccur and that, without professional help, the people described would get worse.

**Conclusions:**

The beliefs that non-mental health professionals hold about mental disorders are inadequate to provide appropriate help. There is an urgent need for mental health education campaigns to improve non-mental health professionals’ mental health knowledge in mainland China in order to provide better support for mental health service users.

## Introduction

Mental health has always been a challenging public health issue. Mental and substance disorders accounted for 6.6% of global disability-adjusted life years and 18.9% of global years lived with disability, making them among the leading causes of the non-communicable disease burden in 2015 [1,2]. In 2013, the total expenditure of mental disorders accounted for over 15% of the total health expenditure in mainland China and for 1.1% of gross domestic product [3]. With the rapid development of the economy and of society, common mental disorders (e.g. anxiety disorder and depression) and psychological behavioral disorders are increasing, leading to severe challenges in mental health work in mainland China. Mental health service resources are insufficient and unevenly distributed. According to the 2015–2020 National Mental Health Work Plan of China there were 1650 specialized mental health institutions, 228,000 psychiatric beds with an average of 1.71 for 100,000 population (4.36 for 100,000 population globally) and over 20,000 psychiatrists nationally; most of these resources were distributed at the provincial or municipal levels, and a community-based rehabilitation system for mental disorders has not yet been established [4]. The public’s awareness of anxiety, depression, and other common mental disorders, as well as about psychological behavioral problems, is poor. Because of the obvious social biases and stigma towards mental illnesses, most mental health service users would rather conceal their illness, with few seeking support from mental professionals [4].

A survey conducted in four provinces in China (2001–2005) showed that the adjusted 1-month prevalence of all mental disorders were 17.5%, and 24% of persons with mental disorders had moderate to severe disability [5]. More than 88% of patients with nonpsychotic mental disorders had never sought any professional help for their problems; among those patients who had sought professional help, 41% had been treated only by surgeons or physicians who practice Western medicine in areas other than mental health, or by practitioners of traditional Chinese medicine [5]. Although non-mental health professionals have constant contact with psychiatric patients, especially non-psychotic patients in their daily clinical practice, their short of knowledge about mental disorders may prevent them from providing appropriate support to mental health service users.

Mental health literacy (MHL) has been defined as “knowledge and beliefs about mental disorders which aid their recognition, management or prevention.”[6] According to this definition, MHL includes: the ability to identify specific disorders; being aware of how to search information; knowledge of related risk factors, causes, self-management, and how to seek available professional help; and having attitudes that promote identification [6]. While much research has been done in Western countries on the MHL of both the public and professionals [7–11], no published data are currently available on the MHL of non-mental health professionals in mainland China, as assessed by vignettes. Most studies investigating MHL have centered on depression and schizophrenia [7,11,12]. These two disorders are investigated in the present study, with the addition of generalized anxiety disorder (GAD) because of its high prevalence across mainland China [5]. Because an understanding of the MHL of health professionals may be helpful in delivery of mental health care, the object of this study was to investigate MHL for schizophrenia, depression, and GAD in a sample of non-mental health professionals enrolled in six general hospitals of Hunan Province in mainland China.

## Methods

### Sample

This study followed a cluster convenience sampling method. Participants were from six general hospitals in the cities of Changsha and Hengyang, Hunan Province, in 2014–2015. These hospitals were the Second Xiangya Hospital of Central South University, the Xiangya Hospital of Central South University, the First Affiliated Hospital of the University of South China, the First People’s Hospital of Hengyang, the Fifth People’s Hospital of Hengyang, and the People’s Hospital of Hengyang County. These hospitals included four tertiary hospitals and two secondary hospitals, all of which are teaching hospitals. No significant regional study was found on the topic and so the sample size was calculated by taking the proportion of knowledge on mental health to be 50%, at a 95% confidence level, 5% marginal error, and adding a 10% non-response rate. The required sample size was 424 for each questionnaire: we chose 450 for convenience, with each hospital having 75 respondents for each questionnaire, and so a total of 1350 questionnaires were sent to participants, who completed the questionnaire on their own.

The protocol was confirmed by the ethics committee of the Second Xiangya Hospital of Central South University. The distributed questionnaires clearly stated the aim of the study and informed the participants that they consented to take part in by filling out the questionnaire.

### Survey questionnaire

The survey questionnaire was revised from that used by Jorm et al. [6] to investigate MHL among Australian samples. To apply a Western study instrument to a Chinese sample, we got in touch with the author of the instrument, and obtained his consent to use the instrument and to make relevant changes if necessary. In the revised questionnaires, additional interventions such as having a massage, Qigong/Tai Chi therapy, and acupuncture were added to fit with Chinese conditions. Interventions used in those Australian study but not suitable for Chinese conditions were removed. The validity of the questionnaire was examined in a previous survey [13].

Participants were randomly assigned to receive one of three vignettes (see S1 File) describing (1) schizophrenia, (2) depression, or (3) GAD. The symptoms described in the vignettes met the diagnostic criteria for schizophrenia, depression, or GAD in both the fourth edition of the *Diagnostic and Statistical Manual of Mental Disorders* and the 10th revision of the *International Statistical Classification of Diseases and Related Health Problems*.

Following the presentation of the vignette, participants were asked what they thought was the most likely diagnosis (with a series of diseases to choose), and the likely helpfulness of various interventions (rated as helpful, harmful, or neither). All answers were single choice. Participants were allowed to choose three answers when asked about how the person in the vignette could best be helped. The questionnaire also included questions about the likely result with and without professional help. Data related to these questions are reported in the Results.

Other questions in the questionnaire included those related to causes and risk factors about disease, stigma, discrimination, and social distance. Data related to these latter questions will be reported elsewhere. Demographic data were also collected, including age, gender, hospital level, educational level, specialty of participants, and years of work.

### Statistical analysis

We entered information by using a double-entry strategy in EpiData, version 3.1 (EpiData Association, Odense, Funen, Denmark). Data were analyzed with IBM-SPSS 20.0 software (IBM Corp., Armonk, NY, USA). In this survey, data analysis was mainly descriptive, with median (interquartile range) used for skewed variables and frequency (percentage) for categorical variables.

## Results

### Demographics

A total of 1123 doctors completed the survey (response rate of 83.26%). The numbers of responses for each of the three vignette types were as following: schizophrenia, n = 377; depression, n = 372; GAD, n = 374. No significant differences were found between vignettes in terms of age, gender, specialty, level of education, years of work, or hospital level. As Table 1 shows, 54.4% of the participants were male. About 78.2% of the participants had a bachelor's degree or higher. The median age of the participants was 28 years, and the median number of working years was 4. About 60% of the participants were physicians, and over 70% were from tertiary hospitals.

**Table 1:** Socio-demographic characteristics of participants by vignette.

Participants characteristics	Schizophrenia vignettes		Depression vignettes		GAD vignettes	
	N = 377		N = 372		N = 374	
	n	(%)	n	(%)	n	(%)
Gender						
Male, n(%)	204	(54.1)	203	(54.6)	204	(54.5)
Female, n(%)	173	(45.9)	169	(45.4)	170	(45.5)
Age(years)*^a^	28	(25–35)	27	(25–35)	28	(25–34.3)
Educational level						
<Bachelor's degree, n(%)	67	(17.8)	70	(18.8)	66	(17.6)
Bachelor's degree, n(%)	174	(46.2)	168	(45.2)	164	(43.9)
Master’s degree, n(%)	119	(31.5)	109	(29.3)	119	(31.8)
Doctor’s degree, n(%)	17	(4.5)	25	(6.7)	25	(6.7)
Specialty						
Physician, n (%)	230	(61.0)	223	(59.9)	223	(59.6)
Surgeon, n (%)	147	(39.0)	149	(40.1)	151	(40.4)
Work duration(years)*^a^	4	(2–10)	4	(2–10)	4	(2–10)
Hospital level						
Tertiary hospital, n (%)	275	(72.9)	273	(73.4)	259	(71.7)
Secondary hospital, n (%)	102	(27.1)	99	(26.6)	102	(28.3)

* ^a^ Data descriptive with median (interquartile range).

GAD, generalized anxiety disorder.

### Identification of disorders

Table 2 shows the frequency of participants who labeled each of the categories describing the problems shown in the vignettes. Schizophrenia was correctly identified by 48.8% (n = 184) of non-mental health professionals for the schizophrenia vignettes, depression correctly identified by 58.1% (n = 216) for the vignettes, and GAD correctly identified by 31.8% (n = 119) for the GAD vignettes.

**Table 2:** Frequency of participants who labeled each problem shown in the vignettes.

Disorders	Schizophrenia vignettes		Depression vignettes		GAD vignettes	
	N = 377		N = 372		N = 374	
	n	(%)	n	(%)	n	(%)
Stress	11	(2.9)	41	(11.0)	38	(10.2)
Depression	33	(8.8)	216	(58.1)	39	(10.4)
Schizophrenia	184	(48.8)	7	(1.9)	10	(2.7)
Psychological problems	12	(3.2)	11	(3.0)	17	(4.5)
Mental illness	51	(13.5)	18	(4.8)	12	(3.2)
Physical illness	3	(0.8)	11	(3.0)	9	(2.4)
Emotional problems	1	(0.0)	19	(5.1)	4	(1.1)
Self-esteem	5	(1.3)	1	(0.3)	0	(0.0)
Spirit intrusion	2	(0.5)	0	(0.0)	0	(0.0)
Phobia	5	(1.3)	0	(0.0)	6	(1.6)
Social phobia	26	(6.9)	7	(1.9)	42	(11.2)
GAD	9	(2.4)	14	(3.8)	119	(31.8)
PTSD	14	(3.7)	3	(0.8)	2	(0.5)
Acute anxiety attack	3	(0.8)	10	(2.7)	23	(6.1)
OCD	1	(0.3)	0	(0.3)	32	(8.6)
Personality disorder	7	(1.9)	1	(2.7)	7	(1.9)
Caused by physical illness	8	(2.1)	10	(0.8)	6	(1.6)
Other	1	(0.3)	3	(1.9)	6	(1.6)
Don’t know	1	(0.3)	0	(0.0)	2	(0.5)

GAD, generalized anxiety disorder; OCD, obsessive-compulsive disorder; PTSD, post-traumatic stress disorder.

### Helpfulness of interventions

Table 3 shows the frequency of participants who rated people and medications as helpful for schizophrenia, depression, and GAD.

**Table 3:** Frequency of participants who rated people and medications as ‘helpful’ in the vignettes.

Interventions	Schizophrenia vignettes		Depression vignettes		GAD vignettes	
	N = 377		N = 372		N = 374	
	n	(%)	N	(%)	n	(%)
**People who can help**						
A typical GP or family doctor	243	(64.5)	240	(64.5)	238	(63.6)
A pharmacist	109	(28.9)	95	(25.5)	115	(30.7)
A counselor	212	(56.2)	223	(59.9)	231	(61.8)
A social worker	140	(37.1)	154	(41.4)	176	(47.1)
A telephone counseling service	135	(35.8)	149	(40.1)	183	(48.9)
A psychiatrist	345	(91.5)	324	(87.1)	334	(89.3)
A psychiatric nurse	289	(76.7)	273	(73.4)	273	(73.0)
A clinical psychologist	337	(89.4)	327	(87.9)	333	(89.0)
Help from close family	316	(83.8)	308	(82.8)	324	(86.6)
Help from close friends	302	(80.1)	305	(82.0)	315	(84.2)
An herbalist	113	(30.0)	126	(33.9)	128	(34.2)
San Zhang/Si Li/Wu Wang can try to deal with his/her problems on his/her own	32	(8.5)	36	(9.7)	46	(12.3)
Pray to Buddha for help	19	(5.0)	25	(6.7)	103	(27.5)
**Medications which can help**						
Vitamins and mineral	115	(30.5)	129	(34.7)	146	(39.0)
Laxatives such as lactulose or senna	40	(10.6)	36	(9.7)	45	(12.0)
Tonics or herbal medicines;	83	(22.0)	79	(21.2)	91	(24.3)
Pain relievers, such as aspirin or acetaminophen	41	(10.9)	49	(13.2)	40	(10.7)
Antidepressants	247	(65.5)	291	(78.2)	228	(61.0)
Antibiotics	38	(10.1)	50	(13.4)	45	(12.0)
Sleeping pills	131	(34.7)	191	(51.3)	173	(46.3)
Antipsychotics	320	(84.9)	216	(58.1)	189	(50.5)
Tranquillizers such as diazepam	233	(61.8)	203	(54.6)	227	(60.7)
Anxiolytics	265	(70.3)	246	(66.1)	297	(79.4)

GAD, generalized anxiety disorder; GP, general practitioner.

A descriptive analysis of persons and interventions that may be helpful showed that the majority of non-mental health professionals agreed that a psychiatrist would be helpful in schizophrenia (91.5%), depression (87.1%), and GAD (89.3%). A clinical psychologist was considered as helpful in 89.4% of participants for schizophrenia, 87.9% for depression, and 89.0% for GAD. Help from close family was rated as helpful by 83.8% of participants for schizophrenia, 82.8% for depression, and 86.6% for GAD. Help from close friends was endorsed by 80.1% for schizophrenia, 82.0% for depression, and 84.2% for GAD. Almost one third of participants (30.0% for schizophrenia, 33.9% for depression, and 34.2% for GAD) endorsed the helpfulness of an herbalist. Praying to Buddha for help was considered helpful in 5% of participants for schizophrenia, 6.7% for depression, and 27.5% for GAD. For the schizophrenia vignettes, antipsychotics were rated as the most helpful (84.9%) medication, followed by anxiolytics and antidepressants. For the depression vignettes, antidepressants were rated as the most helpful (78.2%), followed by anxiolytics and antipsychotics. For the GAD vignettes 79.0% of participants agreed that anxiolytics would be helpful, followed by antidepressants and tranquillizers. Over half of the participants in all vignettes thought tranquillizers such as diazepam would be helpful.

As Table 4 shows, Becoming physically more active, getting out more and psychotherapy were the activities and therapies rated as most helpful for all vignettes. For the schizophrenia vignette, staying at home and resting, electroconvulsive therapy (ECT), and having an occasional drink were considered among the least helpful interventions, whereas for the depression and GAD vignettes, ECT, staying at home and resting and going on a special diet or avoiding certain foods were rated as the least helpful interventions. About half of the participants believed that massage to relax, Qigong/Tai Chi therapy, and acupuncture therapy were helpful in these vignettes.

**Table 4:** Frequency of participants who rated other interventions as ‘helpful’ in the vignettes.

Other Inventions	Schizophrenia vignette		Depression vignette		GAD vignette	
	N = 377		N = 372		N = 374	
	n	(%)	n	(%)	n	(%)
Becoming physically more active, such as playing more sports, or doing a lot more walking or gardening	321	(85.1)	322	(86.6)	326	(87.2)
Reading about people with similar problems and how they have dealt with them	284	(75.3)	292	(78.5)	306	(81.8)
Getting out more	320	(84.9)	335	(90.1)	336	(89.8)
Staying at home and resting	78	(20.7)	117	(31.5)	109	(29.1)
Attending courses or relaxation, stress management, meditation, or yoga	278	(73.7)	293	(78.8)	304	(81.3)
Massage to relax	260	(69.0)	300	(80.6)	305	(81.6)
Cutting out alcohol altogether	223	(59.2)	223	(59.9)	234	(62.6)
Having an occasional alcoholic drink to relax	121	(32.1)	151	(40.6)	165	(44.1)
Qigong/Tai Chi therapy	163	(43.2)	178	(47.8)	202	(54.0)
Acupuncture therapy	155	(41.1)	159	(42.7)	173	(46.3)
Psychotherapy	313	(83.0)	313	(84.1)	322	(86.1)
Hypnosis	241	(63.9)	262	(70.4)	263	(70.3)
Aromatic therapy	168	(44.6)	198	(53.2)	204	(54.5)
Being admitted to a psychiatric ward of a general hospital	298	(79.0)	239	(64.2)	230	(61.5)
Being admitted to a psychiatric hospital	290	(76.9)	214	(57.5)	204	(54.5)
Undergoing electro-convulsive therapy	115	(30.5)	83	(22.3)	76	(20.3)
Going on a special diet or avoiding certain foods.	148	(39.3)	142	(38.2)	138	(36.9)

GAD, generalized anxiety disorder.

### Best method of help

Table 5 shows the frequency of selecting each category about e question about how the participants would help the person in the vignette if he/she was a number of their families or a friend. Participants were allowed to choose three answers. For all vignettes, listening/talking with the person was considered the most helpful. Other common responses were encouraging the person to see a psychologist, accompanying the person to professional help, and encouraging the person to see a psychiatrist.

**Table 5:** Frequency of participants selecting each category about question how the person could best be helped.

Help methods	Schizophrenia vignettes		Depression vignettes		GAD vignettes	
	N = 377		N = 372		N = 374	
	n	(%)	n	(%)	n	(%)
Listen/talk with the person	167	(44.2)	198	(53.2)	202	(54.0)
Accompany the person to professional help	137	(36.2)	130	(34.9)	132	(35.3)
Contact professional help on the person’s behalf	83	(22.0)	57	(15.3)	62	(16.6)
Encourage the person to seek help	85	(22.5)	78	(21.0)	72	(19.3)
Encourage the person to see a community physician	18	(4.8)	16	(4.3)	16	(4.3)
Encourage the person to see a counselor	66	(17.5)	112	(30.1)	102	(27.3)
Encourage the person to see a psychiatrist	95	(25.1)	103	(27.7)	115	(30.7)
Encourage the person to see a psychologist	137	(36.6)	95	(25.5)	91	(24.3)
Encourage the person to contact a helpline	11	(2.9)	5	(1.3)	6	(1.6)
Encourage the person to go to hospital	31	(8.5)	28	(7.5)	22	(5.9)
Encourage the person to go to a mental health clinic	98	(26.3)	62	(16.7)	51	(13.6)
Ask if the person wants help	12	(3.2)	13	(3.5)	19	(5.1)
Assess the problem/risk of harm	26	(6.9)	25	(6.7)	16	(4.3)
Do an intervention	8	(2.1)	5	(1.3)	4	(1.1)
Cheer the person up/boost the person’s confidence	23	(6.1)	34	(9.1)	31	(8.3)
Give advice	16	(4.2)	17	(4.6)	23	(6.1)
Seek information for the person	3	(0.8)	1	(0.3)	7	(1.9)
Help the person make new friends	13	(3.4)	9	(2.4)	9	(2.4)
Help with chores/work	1	(0.3)	3	(0.8)	6	(1.6)
Provide general support (e.g. practical, emotional)	25	(6.6)	37	(9.9)	35	(9.4)
Spend time/socialize with the person	26	(6.9)	40	(10.8)	45	(12.0)
Encourage the person to become physically active	16	(4.2)	25	(6.7)	29	(7.8)
Tell the person’s parents or family	26	(6.9)	20	(5.4)	22	(5.9)

GAD, generalized anxiety disorder.

### Beliefs about outcomes

Table 6 shows the data for beliefs about outcomes with or without professional help. The most common belief was that the person would experience full recovery, but problems would probably reoccur with professional help for all vignettes. For the schizophrenia vignette, 7.9% of participants considered that getting worse was the most likely outcome without professional treatment; whereas less than 50% of participants considered this to be the most likely outcome for the depression and GAD vignettes.

**Table 6:** Frequency of participants choosing each outcome as likely for the person described in the vignette.

Likely outcomes	Schizophrenia vignettes		Depression vignettes		GAD vignettes	
	N = 377		N = 372		N = 374	
	n	(%)	n	(%)	n	(%)
**With professional help**						
Full recovery with no further problems	49	(13.0)	73	(19.6)	70	(18.7)
Full recovery, but problems would probably reoccur	234	(62.1)	252	(67.7)	226	(60.4)
Partial recovery	41	(10.9)	22	(5.9)	36	(9.6)
Partial recovery, but problems would probably reoccur	41	(10.9)	16	(4.3)	27	(7.2)
No improvement	2	(0.5)	2	(0.5)	5	(1.3)
Get worse	10	(2.7)	2	(0.5)	2	(0.5)
Don't know	49	(13.0)	5	(1.3)	8	(2.1)
**Without professional help**						
Full recovery with no further problems	6	(1.6)	18	(4.8)	14	(3.7)
Full recovery, but problems would probably reoccur	25	(6.6)	53	(14.2)	40	(10.7)
Partial recovery	11	(2.9)	19	(5.1)	20	(5.3)
Partial recovery, but problems would probably reoccur	30	(8.0)	63	(16.9)	56	(15.0)
No improvement	14	(3.7)	26	(7.0)	36	(9.6)
Get worse	271	(71.9)	176	(47.3)	184	(49.2)
Don't know	20	(5.3)	17	(4.6)	24	(6.4)

GAD, generalized anxiety disorder.

## Discussion

This is the first study to examine the MHL of non-mental health professionals in mainland China by using vignettes. The survey showed that the rate of identification for the depression vignette was relatively higher than those for the schizophrenia and the GAD vignettes. When the participants were asked about the helpfulness of various people, medications, and interventions, although they tended to give the highest ratings to medical interventions (psychiatrists, clinical psychologists, antipsychotics, antidepressants, anxiolytics, psychotherapy), they also rated lifestyle interventions (help from close family, psychical activity, getting out more) highly.

### Identification of the disorders in the vignettes

The identification rate for depression was the highest among the three vignettes, followed by schizophrenia and GAD, but the rats for all were lower than 60%. This study showed that non-mental health professionals identified depression more easily than they did schizophrenia, which is consistent with most previous findings [7,8,11]. The identification rate for GAD was the lowest, which may be because, compared with GAD, schizophrenia and depression get more attention in books, movies, TV shows, and awareness campaigns [14]. Over 10% of the participants labeled schizophrenia as a mental illness, whereas less than 5% of participants labeled depression or GAD as a mental illness. Over 10% of the participants labeled depression or GAD as stress or labeled GAD as depression. The results showed there would be a high likelihood that non-mental health professionals misdiagnose or miss diagnosing these common mental disorders. This finding is worrying, as non-mental health professionals have constant contact with patients and thus have a potential role in screening and referring people to the appropriate care when there are early warning signs of mental illnesses. For anxiety and mood disorders, it can take long time between the onset of these disorders and the introduction of appropriate professional help. One study showed that most people (71%) with anxiety or mood disorder reported that they first sought help from a general medical practitioner, with at least 1-month delay [15]. Another study showed that the misdiagnosis rate reached 65.9% for major depressive disorder and 71.0% for GAD [16]. Patients with GAD utilize primary care resources frequently, and many of them visit a quantity of physicians before they are correctly diagnosed and treated. This approach is related to a significant economic burden due to decreased work efficiency and increased utilization of health care services, specifically primary health care [17]. A high level of MHL would make early identification of these disorders and referral to mental health specialists more likely, which could help reduce the burden of the diseases.

### Helpfulness of interventions

For all vignettes, non-mental health professionals were more likely to rate psychiatrists, clinical psychologists, close family, and close friends as being more helpful than others. Beliefs about the helpfulness of different people for the three vignettes were almost the same, except that 27.5% of participants believed praying to Buddha would be helpful in the GAD vignettes, which may be related to failure to recognize GAD as a mental illness and to label it as stress or depression. The results also showed that non-mental health professionals tended to prefer seeking help from other specialists than from general health practitioners, which is contrary to the public’s belief [10]. About one third of participants thought herbalists were helpful, which is related to Chinese traditional culture. The ratings for herbalists as being helpful were higher than those for psychiatrists’ in the study conducted by Liu et al. [13].

When asked about the helpfulness of a wide range of medications, participants gave the highest ratings to antipsychotics for schizophrenia, to antidepressants for depression, and to anxiolytics for GAD. Over 50% of participants believed that antidepressants, antipsychotics, tranquillizers, and anxiolytics were helpful in all vignettes. The belief of non-mental health professionals in the helpfulness of psychotropic drugs for different mental disorders spread to the treatment of other conditions. Over one third rated sleeping pills as being helpful for all disorders. The ratings for these medications were higher compared to those reported for the public’s beliefs in Australia and Japan [18]. Although over 80% of the participants in the current study believed that psychotherapy was helpful in all disorders, they gave higher helpfulness ratings to lifestyle interventions (e.g. getting out more, physical activity, reading books about people with similar problems to learn the way they have dealt with them, and relaxation) than to those professional help (e.g. undergoing ECT). Over 75% of participants rated being admitted to a psychiatric ward of a general hospital, and being admitted to a psychiatric hospital, as helpful for schizophrenia, whereas fewer rated these admissions as being helpful for depression and GAD, especially being admitted to a psychiatric hospital. Perhaps this was because participants did not consider depression and GAD as mental illnesses and had no idea about ECT.

The findings suggested that non-mental health professionals had almost the same belief systems for the treatment of schizophrenia, depression, and GAD. However, there were differences in other interventions among the three vignettes. Lifestyle treatments, such as becoming more physically active and getting out more, were rated more positively for depression and GAD than for schizophrenia, and the belief systems for depression and GAD were closer, which may be because many participants labeled depression and GAD as stress and depression. Psychological interventions, such as visiting a social worker, telephone counseling, and hypnosis, had higher ratings for depression and GAD than for schizophrenia. This finding suggests that participants would advise patients to change their lifestyle first, rather than visit mental health professionals.

### Best method of help

Although participants rated psychiatrists and clinical psychologists as being more helpful than close family and close friends for all vignettes, the rating for listening/talking with the person was the highest among all the methods of help in all vignettes. Although participants believed they should accompany the person to professional help in all disorders, they rated psychologists most highly for schizophrenia, counsellors for depression, and psychiatrists for GAD. This result suggests that participants believed that all disorders in the vignettes may be settled with the help of family and friends’ first.

### Beliefs about outcomes

For all vignettes, most non-mental health professionals shared the same belief about outcomes from professional treatment. Over 70% of participants believed that, without help, the person in the schizophrenia vignette would get worse, whereas less than 50% of participants believed that the person in the depression and the GAD vignettes would get worse. This result may suggest that about 50% of participants believe patients with depression and GAD would get better through other non-professional interventions, such as getting out more, and becoming physically more; participants may prefer changing their lifestyle when faced with the same problem. This finding is consistent with the participants’ identification of the disorders.

The survey showed that the MHL of non-mental health professionals was not ideal. Although participants thought that some psychiatric interventions (psychiatrists) and psychological interventions (psychologists and psychotherapy) were helpful, lifestyle interventions (help from close family and close friends, becoming physically more active, getting out more) were also rated highly in all vignettes, especially for depression and GAD. Some psychiatric interventions, such as ECT and being admitted to a psychiatric ward, were thought to be less helpful than some lifestyle interventions. This may explain part of the mental health conditions in China.

The total expenditure of mental illnesses in 2013 has been estimated to account for over 15% of the total health expenditure in China and for 1.1% of gross domestic product [3]. If the professional care needs for all patients with mental illnesses were adequately fulfilled, the potential economic expenditure would achieve up to 6 times [3]. In mainland China, general physicians, surgeons, and primary-care health workers usually receive little or no mental health training, which makes them unable (and usually unwilling) to provide basic mental health services [5]. Non-mental health professionals in general hospitals study knowledge about mental illness on their own rather than learning about it during their formal education [19]. The National Mental Health Work Plan of China (2015–2020) indicated that the level of awareness about psychological well-being among community members in urban and rural areas should reach 70 and 50%, respectively, by 2020 [4]. The work plan also pointed out that general hospitals and other specialized hospitals should provide guidance about psychological well-being to their patients and that primary healthcare facilities should provide education about psychological well-being to residents living in their jurisdictions [4]. Investigation into the factors related to MHL has found that higher education [20,21] and younger age [20,22] are associated with higher MHL. As the participants in the current study have a higher education than do members of the public, there is a long way to go to achieve this goal. The education department should strengthen the education and training of professions related to mental health and ensure that sufficient classroom hours are provided in psychiatry, medical psychology, and related courses in the undergraduate medical curriculum. Health and family planning departments should strengthen and standardize training for residents, develop psychiatric qualification training programs for licensed clinicians or general physicians, and increase their financial input.

This study has several limitations related to the methodology of the survey. Convenience cluster sampling has a higher sampling error, making the survey less representative, although this can also explain the general nature and characteristics of the population to some extent. The study did not discuss the relationships between participants’ specialties and MHL levels, the young cluster and with the experienced cluster, and cognition of the disease and the method chosen for seeking help. These limitations are a result of our analysis method.

## Conclusions

We found that the identification rate for schizophrenia, depression, and GAD among non-mental health professionals in the six general hospitals in Hunan Province was low and that their beliefs about treatment were inadequate to reduce the disease burden caused by mental illness. The findings stress the need to improve non-mental health professionals’ MHL so that they are able to identify mental disorders, take appropriate actions when needed, and provide well-founded support and advice to patients in need.

## Supporting information

S1 File Vignettes describe schizophrenia, depression and GAD. **The schizophrenia vignette**:
San Zhang is 24 and lives at home with his parents. He has had a few temporary jobs since finishing school but is now unemployed. Over the last six months he has stopped seeing his friends, and has begun locking himself in his bedroom and refusing to eat with the family or to have a bath. His parents also hear him walking about in his bedroom at night while they are in bed. Even though they know he is alone, they have heard him shouting and arguing as if someone else is there. When they try to encourage him to do more things, he whispers that he won’t leave home because he is being spied upon by the neighbors. They realize he is not taking drugs because he never sees anyone or goes anywhere.
**The depression vignette**:
Si Li is 26 years old. She has been feeling unusually sad and miserable for the last few weeks. Even though she is tired all the time, she has trouble sleeping nearly every night. Si Li doesn’t feel like eating and has lost weight. She can’t keep her mind on her work and puts off making any decisions. Even day-to-day tasks seem too much for her. This has come to the attention of Li’s boss who is concerned about her lowered productivity.
**The GAD vignette**:
Wu Wang is 45 years old and she is often worried. She worries a great deal about her job performance, her children’s well-being, and her relationships with men. In addition, she worries about a variety of minor matters such as getting to appointments on time, keeping her house clean, and maintaining regular contact with family and friends. It takes Wu Wang longer than necessary to accomplish tasks because she worries about making decisions. Wu Wang has trouble sleeping at night and finds that she is exhausted during the day and irritable with her family.
(DOCX)
