# Supplementary material for: Mental health literacy survey of non-mental health professionals in six general hospitals in Hunan Province of China
Source: PLoS One. 2017 Jul 5;12(7):e0180327. doi: 10.1371/journal.pone.0180327 (PMC5498045; doi:10.1371/journal.pone.0180327)
Supplement: S1 File — The schizophrenia vignette: San Zhang is 24 and lives at home with his parents. He has had a few temporary jobs since finishing school but is now unemployed. Over the last six months he has stopped seeing his friends, and has begun locking himself in his bedroom and refusing to eat with the family or to have a bath. His parents also hear him walking about in his bedroom at night while they are in bed. Even though they know he is alone, they have heard him shouting and arguing as if someone else is there. When they try to encourage him to do more things, he whispers that he won’t leave home because he is being spied upon by the neighbors. They realize he is not taking drugs because he never sees anyone or goes anywhere. The depression vignette: Si Li is 26 years old. She has been feeling unusually sad and miserable for the last few weeks. Even though she is tired all the time, she has trouble sleeping nearly every night. Si Li doesn’t feel like eating and has lost weight. She can’t keep her mind on her work and puts off making any decisions. Even day-to-day tasks seem too much for her. This has come to the attention of Li’s boss who is concerned about her lowered productivity. The GAD vignette: Wu Wang is 45 years old and she is often worried. She worries a great deal about her job performance, her children’s well-being, and her relationships with men. In addition, she worries about a variety of minor matters such as getting to appointments on time, keeping her house clean, and maintaining regular contact with family and friends. It takes Wu Wang longer than necessary to accomplish tasks because she worries about making decisions. Wu Wang has trouble sleeping at night and finds that she is exhausted during the day and irritable with her family. (DOCX) [file pone.0180327.s001.docx]

Supporting Information

S1 File. Vignettes describe schizophrenia, depression and GAD.

**The schizophrenia vignette:**

San Zhang is 24 and lives at home with his parents. He has had a few temporary jobs since finishing school but is now unemployed. Over the last six months he has stopped seeing his friends, and has begun locking himself in his bedroom and refusing to eat with the family or to have a bath. His parents also hear him walking about in his bedroom at night while they are in bed. Even though they know he is alone, they have heard him shouting and arguing as if someone else is there. When they try to encourage him to do more things, he whispers that he won’t leave home because he is being spied upon by the neighbors. They realize he is not taking drugs because he never sees anyone or goes anywhere.

**The depression vignette:**

Si Li is 26 years old. She has been feeling unusually sad and miserable for the last few weeks. Even though she is tired all the time, she has trouble sleeping nearly every night. Si Li doesn’t feel like eating and has lost weight. She can’t keep her mind on her work and puts off making any decisions. Even day-to-day tasks seem too much for her. This has come to the attention of Li’s boss who is concerned about her lowered productivity.

**The GAD vignette:**

Wu Wang is 45 years old and she is often worried. She worries a great deal about her job performance, her children’s well-being, and her relationships with men. In addition, she worries about a variety of minor matters such as getting to appointments on time, keeping her house clean, and maintaining regular contact with family and friends. It takes Wu Wang longer than necessary to accomplish tasks because she worries about making decisions. Wu Wang has trouble sleeping at night and finds that she is exhausted during the day and irritable with her family.
